# Protective Effect of S-Methylcysteine Against *Toxoplasma gondii*-Induced Reproductive Toxicity in Female Albino Rats

**DOI:** 10.1007/s11686-025-01172-2

**Published:** 2025-12-16

**Authors:** Nermeen I. Ashry, Dina M. M. EL Shewehy, Dina A. Elbadry, Amira Ismail

**Affiliations:** 1 Comparative Anatomy and Embryology Department, Faculty of Science, Mansoura University, Al Mansurah, Egypt; 2 Zoology Department, Faculty of Science, Mansoura University, Al Mansurah, Egypt; 3 Department of Medical Parasitology, Faculty of Medicine, Mansoura University, Al Mansurah, Egypt

**Keywords:** *Toxoplasma gondii*, S-Methylcysteine, Spiramycin, Reproductive toxicity, Histopathology

## Abstract

**Background:**

*Toxoplasma gondii* (*T. gondii*) is a zoonotic parasite that causes severe disease, particularly in immunocompromised individuals. Current treatments have significant side effects and are ineffective against the latent stage of the parasite. S-Methylcysteine (SMC), a compound from garlic, has antioxidant and anti-inflammatory properties, but its efficacy against *T. gondii* is unknown.

**Objective:**

To evaluate the in vivo effects of SMC on *T. gondii* infection, focusing on hormonal and histopathological changes and its therapeutic potential.

**Materials and Methods:**

Forty-eight adult female albino rats were divided into eight groups of six. Four groups were infected intra-vaginally with 200 *T. gondii* cysts, while controls received saline. For two months, treatment groups were administered daily doses of SMC (50 mg/kg), Spiramycin (200 mg/kg), or a combination of both via gastric tube. After sacrifice, blood samples were collected for hormonal analysis (estrogen, progesterone, LH, FSH). The ovaries and uterus were excised and histologically examined using hematoxylin and eosin staining for microscopic evaluation.

**Results:**

*Toxoplasma gondii* infection caused significant hormonal disruption and severe uterine and ovarian inflammation. Treatment with either SP or SMC alone partially mitigated these effects. However, the combined SMC and Spiramycin treatment showed the most significant improvement, significantly mitigated hormonal imbalances and promoted substantial tissue recovery.

**Conclusion:**

S-Methylcysteine shows promising therapeutic potential in ameliorating the hormonal and histopathological damage associated with *T. gondii* infection, especially when combined with Spiramycin. Future studies, including human clinical trials, are needed to investigate its mechanisms and confirm its safety and effectiveness.

## Introduction

*Toxoplasma gondii* is a substantial zoonotic parasite of the phylum Apicomplexa, which infects immunocompromised individuals and a wide range of animals, causing severe diseases in hosts [1]. This obligate intracellular parasite is one of the most popular pathogens globally [2]. It is estimated that about one-third of the world's population is chronically infected with *T. gondii*, leading to a significant public health challenge [3]. A recent meta-analysis from Saudi Arabia, continue to modulate these figures, addressing variations in prevalence based on variable risk factors and geographical location [4]. Felids are the definitive hosts, while other warm-blooded animals, such as humans, livestock, marine mammals, and birds, are intermediate hosts [5].

Ingesting tissue cysts in undercooked meat or contact with highly infectious oocysts found in feline feces are the main causes of human infection. Oocysts can also contaminate food supplies and water sources, causing further risks [6]. Organ transplantation and vertical transmission during pregnancy are also less common transmission routes [7]. Various environmental, sociodemographic, and host immune factors affect this parasite's transmission, dissemination, and infection patterns significantly [3].

The parasite exists in three infectious stages: sporozoites (within oocysts), tachyzoites (rapidly multiplying forms), and bradyzoites (tissue cyst forms). Tachyzoites are primarily responsible for clinical manifestations and the acute phase of toxoplasmosis [8]. Upon cell invasion, tachyzoites form a parasitophorous vacuole (PV) for intracellular proliferation, initiating acute infection. In response to host immune pressure, tachyzoites transform into bradyzoites, persisting as latent tissue cysts, predominantly in the brain, nerves, and muscles. These cysts facilitate transmission to new hosts through predation [9].

Acute *T. gondii* infection acquired during pregnancy poses a severe risk of mother-to-child transmission, potentially resulting in spontaneous abortion, stillbirth, or serious postnatal complications [10]. The parasite's ability to establish chronic infection is pivotal for its survival and transmission, yet this stage remains poorly understood in both animal and human hosts [7].

Pyrimethamine and sulfadiazine are the primary pharmacological treatments for toxoplasmosis at this time. Yet, significant side effects, like neutropenia, leukopenia, thrombocytopenia, elevated serum creatinine, hepatic enzyme abnormalities, and hypersensitivity reactions are the main side effects [11]. Azithromycin, clarithromycin, spiramycin, dapsone, atovaquone and cotrimoxazole are alternative drugs that have been used with limited success. These drugs are ineffective and less tolerated against the bradyzoite form of the parasite [12]. Therefore, a global priority for managing toxoplasmosis is evolving more effective and safer treatments [13, 14], especially in the absence of a reliable vaccine which underlines the need for innovative therapeutic approaches [15] though promising progress in vaccine development is continuously being investigated [16].

S-Methylcysteine (SMC) is a sulfur-containing amino acid. Legumes and certain vegetables, including garlic, are rich in this sulfur storage compound [17], which demonstrated diverse therapeutic effects, including anticancer, antioxidant [18, 19], anti-inflammatory, anti-infective [20], antidiabetic, neuroprotective [21, 22], cardioprotective, hepatoprotective [18], and hypocholesterolemic properties [23]. However, the antiprotozoal effects of SMC are still poorly understood [17].

A previous study investigated the efficacy of SMC against *Cryptosporidium parvum* in vivo and reported promising therapeutic potential [18], yet its effects on *T. gondii* remained largely unexplored. To the authors' knowledge, the present study is among the first to specifically examine the in vivo effects of SMC against *T. gondii-*induced reproductive toxicity in a female albino rat model, thus contributing a novel perspective to the understanding of potential therapeutic interventions for toxoplasmosis [24].

## Materials and Methods

### Ethical Approval

All experimental procedures involving animals were conducted in strict accordance with the guidelines for the care and use of laboratory animals and were approved by the Animal Care and Use Committee (ACUC) of Mansoura University, under approval code number MU-ACUC (SC.R.25.07.32). Every effort was made to minimize animal suffering and to reduce the number of animals used.

### Experimental Animals

This study included 48 adult female albino rats (*Rattus norvegicus*) weighing 130–150 g. The animals were obtained from the Theodor Bilharz Research Institute, Giza, Egypt. All rats were acclimatized in the experimental animal house for one week under controlled conditions of 23 ± 2 °C with a 12-h light/dark cycle. The animals were provided with sufficient food and water during the acclimation period. They were randomly divided into eight groups, each containing six rats, using a computer-generated randomization sequence to minimize bias. Four of these groups were infected intra-vaginally (IV) with 200 tissue cysts of *Toxoplasma gondii* (ME-49 strain), representing the infected groups. The ME-49 strain was chosen due to its well-established ability to form tissue cysts and establish chronic infection, which is crucial for studying the latent stage of toxoplasmosis and evaluating therapeutic interventions targeting this stage [25]. To ensure objectivity, investigators performing hormonal assays and histopathological evaluations were blinded to the treatment groups.

### Procedure for Experimental Induction of Parasite

The chronic *Toxoplasma gondii* ME-49 strain was obtained from the Parasitology Department at the Theodor Bilharz Research Institute. The parasite was maintained in the cyst (infective stage) within the brains of chronically infected male albino rats. To isolate the cysts, the infected male rats were sacrificed by cervical dislocation, and their brains were excised. Using glass tissue grinders, the brain tissues were homogenized in 4.0 ml of saline. The cysts were then counted under a compound microscope at 100 × magnification [26].

Each rat in the experimental groups was injected intra-vaginally with 300 µl of saline solution containing 200 cysts of *T. gondii*. In contrast, control rats were injected with an identical volume of normal saline solution [26].

### Chemicals

S-methylcysteine (SMC): Purchased from Sigma-Aldrich (Cairo, Egypt).

Spiramycin 3 MIU: Purchased from Tarshouby Pharmacy (Mansoura, Egypt).

### Experimental Design

Throughout the experiment, the rats were maintained under proper care with adequate food and water. Following the acclimation period, the animals were divided into eight groups (6 rats per group), categorized as either control or infected groups.

The dosages for S-methylcysteine (50 mg/kg body weight) and Spiramycin (200 mg/kg body weight) were selected based on previous studies demonstrating their efficacy and safety profiles in rodent models for similar parasitic infections or related conditions [27, 28]. The two-month treatment duration was chosen to ensure sufficient time for therapeutic effects to manifest and to address the chronic nature of *T. gondii* infection.

#### Control Groups

1. Negative Control Group (C): Injected intra-vaginally with 300 µl of normal saline solution.
2. SMC Group (SMC 50): Administered 1 ml of S-methylcysteine (50 mg/kg body weight) daily via gastric tube for 2 months**.**
3. SP Group: Administered 1.5 ml of Spiramycin 3 MIU (200 mg/kg body weight) daily via gastric tube for 2 months.
4. Combined Treatment Group (SMC + SP): Administered both 1 ml of S-methylcysteine and 1.5 ml of Spiramycin daily for 2 months.

#### Infected Groups

5. Positive Control Group (I): Injected intra-vaginally with 300 µl of suspension containing 200 cysts of *T. gondii* and left untreated.
6. Infected + SMC Group (SMC 50): Administered 1 ml of S-methylcysteine (50 mg/kg body weight) daily via gastric tube for 2 months.
7. Infected + SP Group: Administered 1.5 ml of Spiramycin 3 MIU (200 mg/kg body weight) daily via gastric tube for 2 months.
8. Infected + Combined Treatment Group (SMC + SP): Administered both 1 ml of S-methylcysteine and 1.5 ml of Spiramycin daily for 2 months.

### Sample Collection and Analysis

At the end of the experiment, all rats were sacrificed using a sharp razor blade. Blood samples were collected for hormonal analysis. Organs of the genital system, including the ovary, uterus, and vagina, were excised, and fixed in 10% neutral buffered formalin for histological examination.

#### Hormonal Analysis

Blood samples were collected from each subgroup immediately after sacrificing the animals using a sharp razor blade. The blood was transferred into non-heparinized tubes and centrifuged at 3000 rpm for 5 min to separate the serum. Hormonal levels of progesterone, estrogen, luteinizing hormone (LH), and follicle-stimulating hormone (FSH) were then measured.

#### Histological Study

The uterus and right ovary from all experimental animals were excised, washed with saline, and fixed in 10% neutral buffered formalin for 48 h. Fixed tissues were processed through a series of ascending ethanol concentrations, followed by rinsing in xylene. Paraffin blocks were prepared according to Bancroft and Stevens' method. Thin paraffin Sects. (5 µm) were cut using a microtome and stained with hematoxylin and eosin (H&E) for histopathological examination under a light microscope [29].

### Statistical Analysis

Data from the morphometric and hormonal studies were statistically analyzed using a one-way ANOVA test. Results were presented as mean ± standard deviation (SD), and statistical significance was determined.

## Results

### Serum Estrogen Levels

The results for serum estrogen levels in Table 1 show a significant increase in the infected group (I, 137 ± 1.49 Pg/ml, p < 0.05) compared to the control group (C, 104 ± 2.43 Pg/ml), indicating that *Toxoplasma gondii* infection disrupts hormonal balance, leading to elevated estrogen levels. Treatment with Spiramycin (I SP, 124 ± 1.23 Pg/ml) and S-methylcysteine (I SM, 126 ± 1.34 Pg/ml) significantly reduced estrogen levels compared to the infected group, although they remained higher than control levels. The combined treatment group (I SP SM, 115 ± 2.83 Pg/ml,* p* < 0.05) demonstrated the greatest reduction in estrogen levels among the treated groups, showing significant improvement relative to the infected group. The SP, SM, and SP SM groups (107 ± 2.60, 108 ± 1.77, and 106 ± 2.81 Pg/ml, respectively) exhibited no significant differences compared to the control group, indicating no adverse effects on estrogen levels in healthy animals.

**Table 1:** Serum estrogen (Pg/ml), progesterone (ng/ml), FSH (mIU/ml) and LH level (mIU/ ml) in control and different treated groups

Animal groups	Estrogen		Progesterone		FSH		LH	
	Mean	± SD	Mean	± SD	Mean	± SD	Mean	± SD
C	104	± 2.43	7.7	± 0.262	4.51	± 0.199	12.0	± 0.212
SP	107	± 2.60	7.72	± 0.216	4.44	± 0.174	11.8	± 0.185
SM	108	± 1.77	7.74	± 0.168	4.43	± 0.197	11.7	± 0.222
SP SM	106	± 2.81	7.71	± 0.190	4.46	± 0.204	11.9	± 0.160
I	137	± 1.49^a^	12.2	± 0.338^a^	2.59	± 0.188^a^	9.0	± 0.261^a^
I SP	124	± 1.23^ab^	10.5	± 0.229^ab^	3.52	± 0.131^ab^	10.2	± 0.182^ab^
I SM	126	± 1.34^ab^	10.6	± 0.312^ab^	3.51	± 0.150^ab^	10.1	± 0.142^ab^
I SP SM	115	± 2.83^ab^	9.13	± 0.305^ab^	3.98	± 0.0389^b^	11.0	± ± 0.376^b^

Results are presented as means and ± SD (n = 5 for each group)

^a,b^significant changes at *p* < 0.05

^a^significant as compared to Control group

^b^significant as compared to Infected group

*C *Control, *SP* Spiramycin, *SM* S-Methylcysteine, *I* Infected, *I SP* Infected + Spiramycin, *I SM* Infected + S-Methylcysteine, *I SP SM* Infected + Spiramycin + S-Methylcysteine

### Serum Progesterone Levels

The results for serum progesterone levels in Table (1) indicate a significant increase in the infected group (I, 12.2 ± 0.338 mIU/ml, p < 0.05) compared to the control group (C, 7.70 ± 0.262 mIU/ml), suggesting that *Toxoplasma gondii* infection disrupts hormonal regulation, leading to elevated progesterone levels. Treatment with Spiramycin (I SP, 10.5 ± 0.229 mIU/ml) and S-methylcysteine (I SM, 10.6 ± 0.312 ng/ml) significantly reduced progesterone levels compared to the infected group (I SP, 10.5 ± 0.229 mIU/ml), though they remained higher than control values. The combined treatment group (I SP SM, 9.13 ± 0.305 mIU/ml, *p* < 0.05) demonstrated the greatest improvement, showing a more pronounced reduction in progesterone levels compared to the infected group. The SP, SM, and SP SM groups (7.72 ± 0.216, 7.74 ± 0.168, and 7.71 ± 0.190 ng/ml, respectively) exhibited no significant changes compared to the control group, indicating that these treatments do not alter progesterone levels in healthy animals.

### Serum Follicle-Stimulating Hormone Levels

The results for serum follicle-stimulating hormone (FSH) levels in Table (1) show a significant decrease in the infected group (I, 2.59 ± 0.188 mIU/ml, *p* < 0.05) compared to the control group (C, 4.51 ± 0.199 mIU/ml), indicating that *Toxoplasma gondii* infection disrupts normal hormonal regulation and reduces FSH levels. Treatment with Spiramycin (I SP, 3.52 ± 0.131 mIU/ml) and S-methylcysteine (I SM, 3.51 ± 0.150 mIU/ml) significantly improved FSH levels compared to the infected group, though they remained lower than control values. The combined treatment group (I SP SM, 3.98 ± 0.0389 mIU/ml, * p* < 0.05) exhibited the greatest improvement, showing a significant increase compared to the infected group, nearing control levels. The SP, SM, and SP SM groups (4.44 ± 0.174, 4.43 ± 0.197, and 4.46 ± 0.204 mIU/ml, respectively) displayed no significant changes compared to the control group, indicating that these treatments did not affect FSH levels in healthy animals.

### Serum Luteinizing Hormone Levels

The results for serum luteinizing hormone (LH) levels in Table 1 show a significant decrease in the infected group (I, 9.0 ± 0.261 ng/ml, *p* < 0.05) compared to the control group (C, 12.0 ± 0.212 ng/ml), indicating hormonal disruption caused by *Toxoplasma gondii* infection. Treatment with Spiramycin (I SP, 10.2 ± 0.182 ng/ml) and S-methylcysteine (I SM, 10.1 ± 0.142 mIU/ml) significantly improved LH levels compared to the infected group, although they remained below control values. The combined treatment group (I SP SM, 11.0 ± 0.376 mIU/ml, *p* < 0.05) demonstrated the greatest improvement, with LH levels approaching control values. The SP, SM, and SP SM groups (11.8 ± 0.185, 11.7 ± 0.222, and 11.9 ± 0.160 mIU/ml, respectively) showed no significant changes compared to the control group, confirming no adverse effects on LH levels in healthy animals.

### Histopathological Examination of the Uterus in Control, Infected, and Treated Groups

**Fig. 1:**
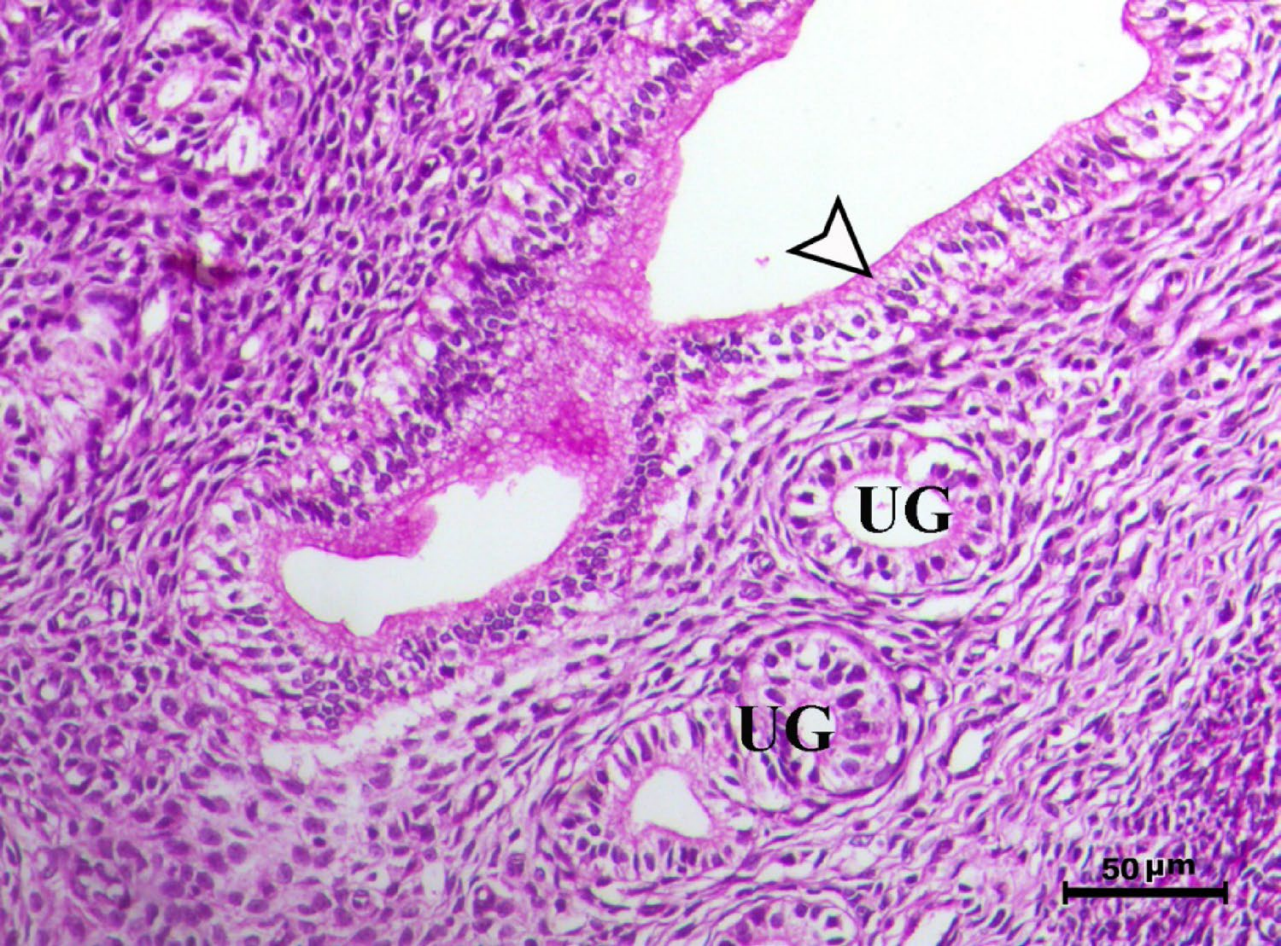
Uterus of control animal G1 showing normal endometrial lining epithelium (arrowhead) and normal endometrial glands (UG), H&E stain, bar = 50 µm

**Fig. 2:**
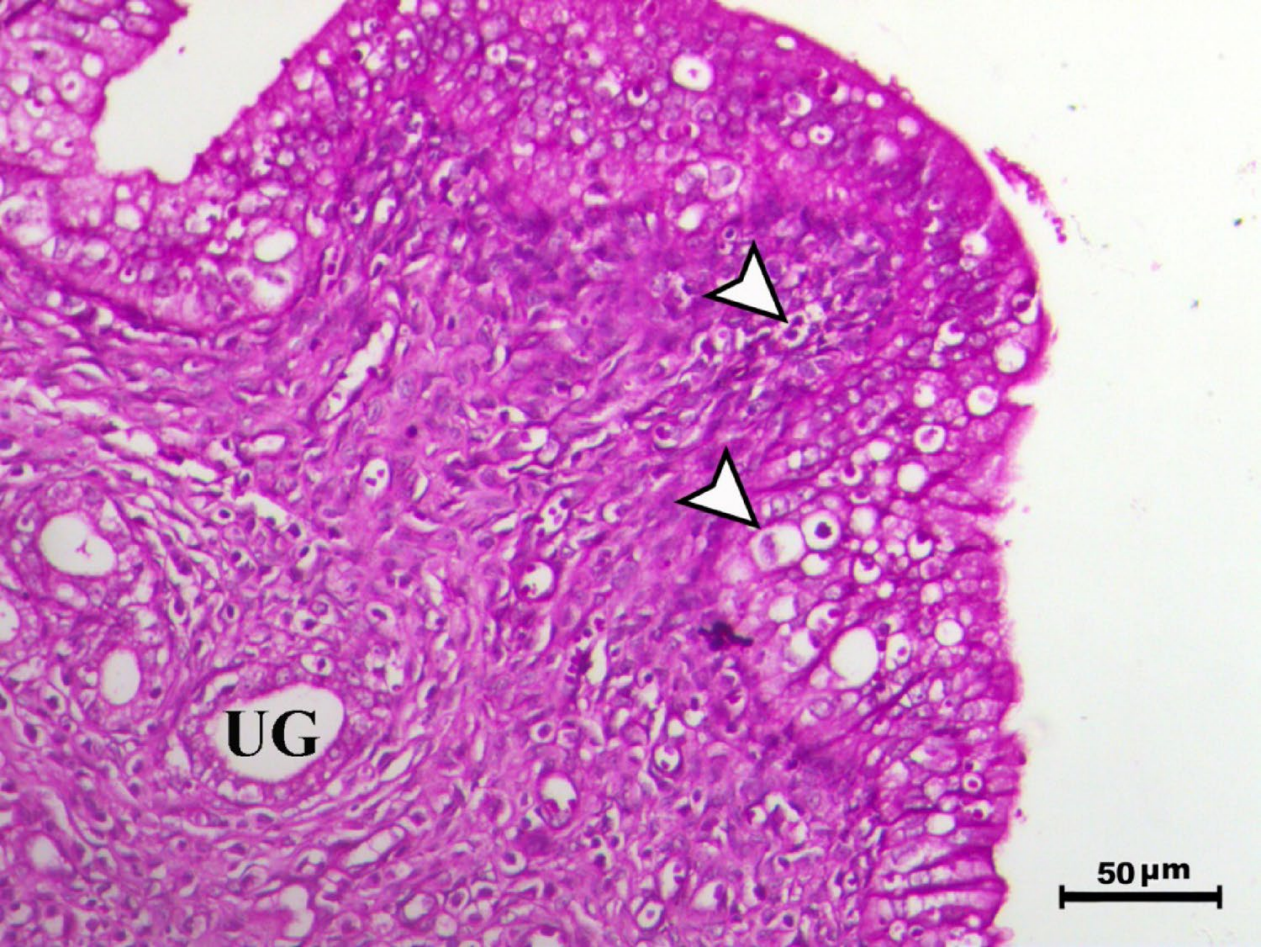
Uterus of diseased animal G2 showing catarrhal endometritis associated with hyperplasia of the endometrial epithelium with presence of the toxoplasma cyst (arrowhead) (UG indicates endometrial glands), H&E stain, bar = 50 µm

**Fig. 3:**
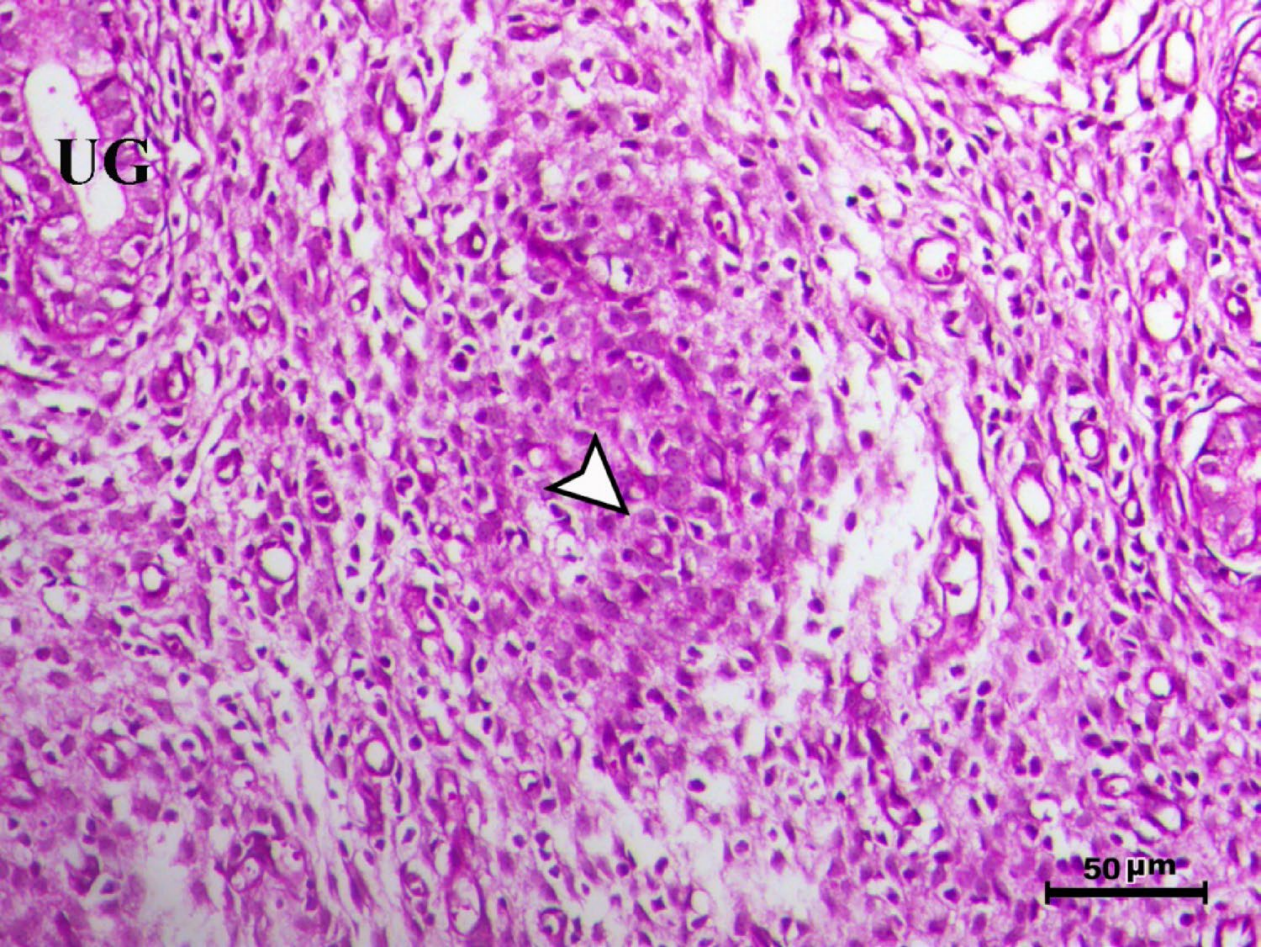
Uterus of diseased animal G2 showing granuloma-like lesions within the endometrial mucosa associated with mononuclear cells infiltration (arrowhead) (UG indicates endometrial glands), H&E stain, bar = 50 µm

**Fig. 4:**
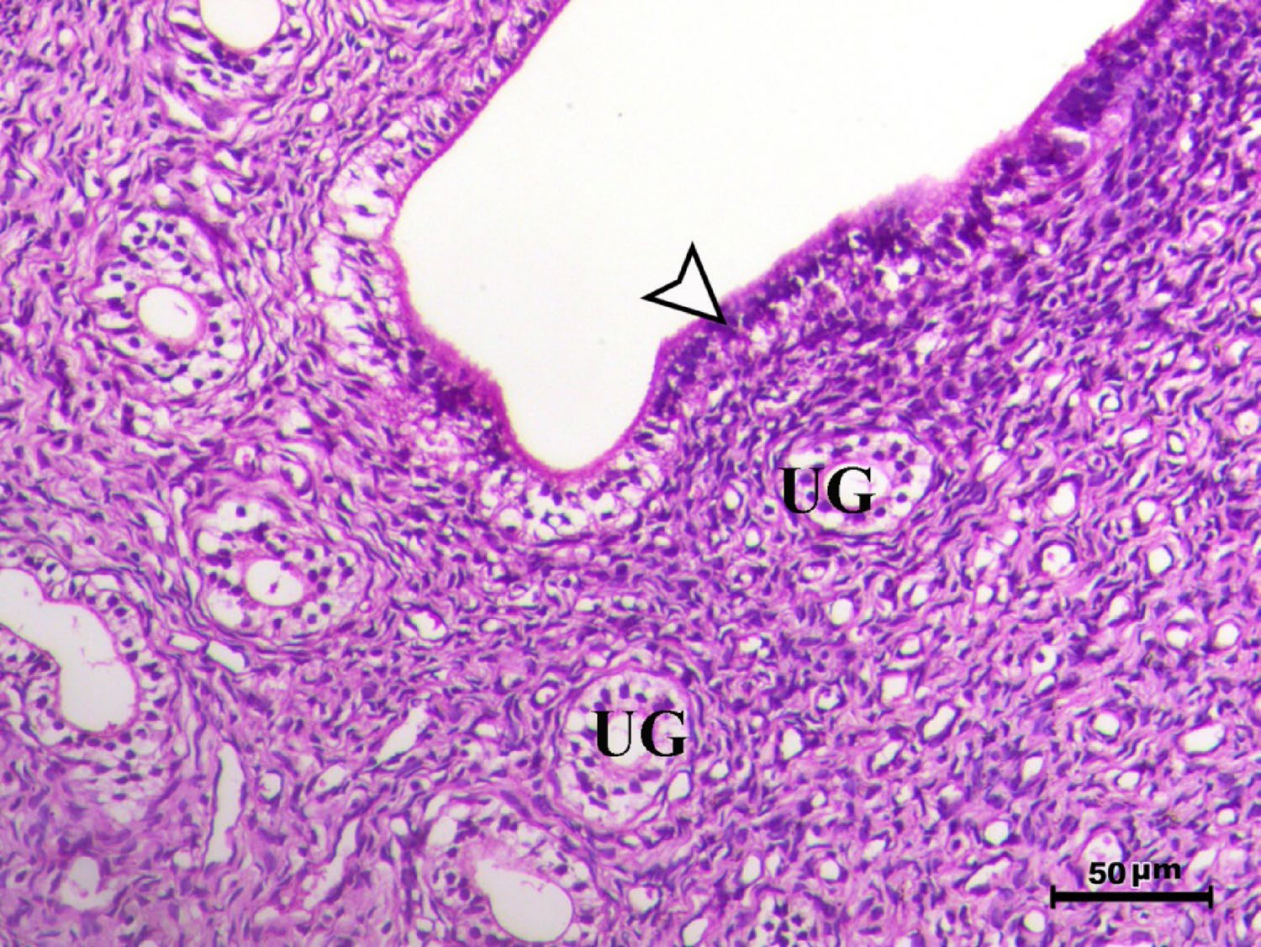
Uterus of control animal G3 showing normal endometrium with normal endometrial lining (arrowhead) and normal endometrial glands (UG), H&E stain, bar = 50 µm

**Fig. 5:**
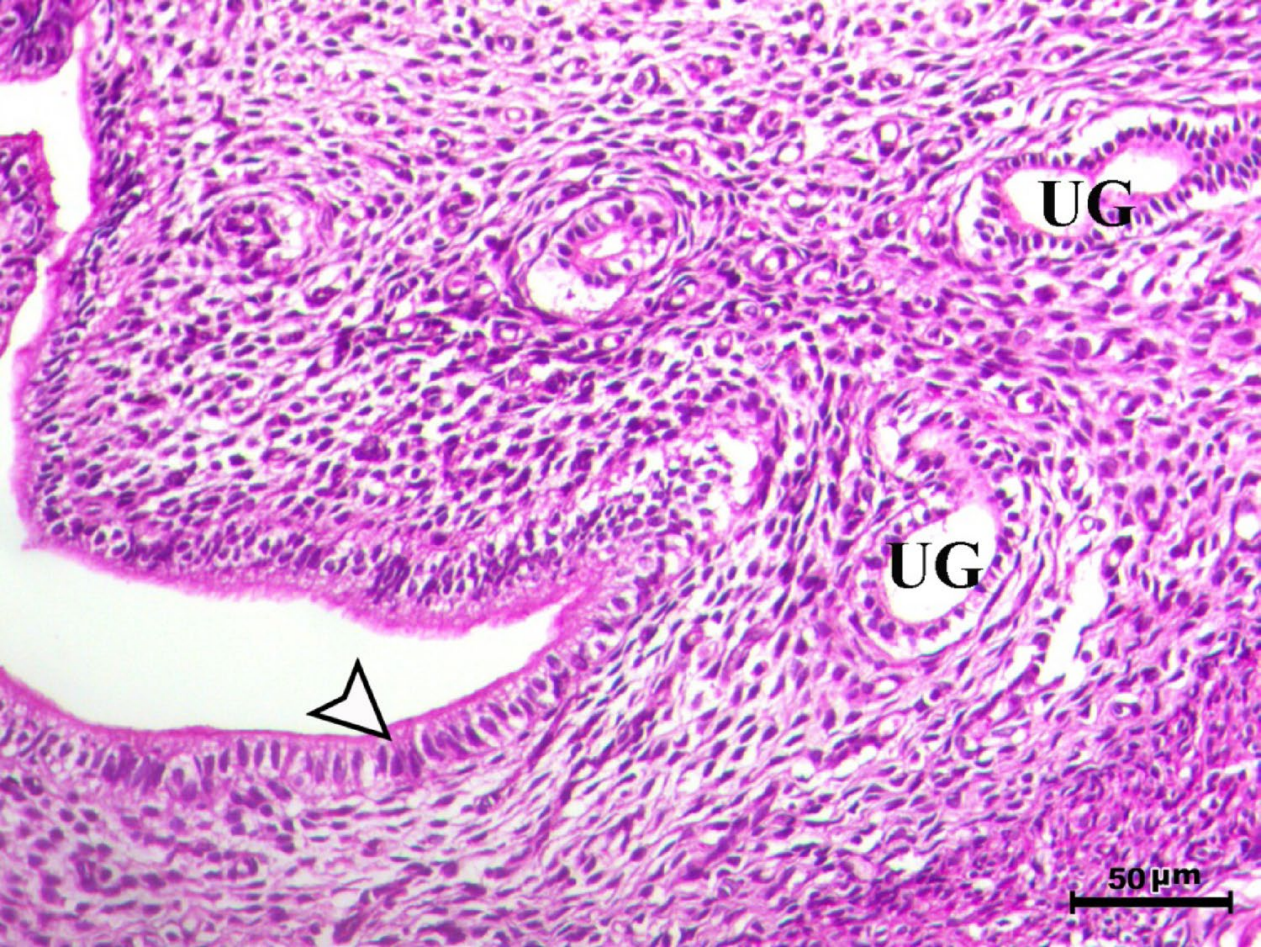
Uterus of control animal G4 showing normal endometrium with normal endometrial lining (arrowhead) and normal endometrial glands (UG), H&E stain, bar = 50 µm

**Fig. 6:**
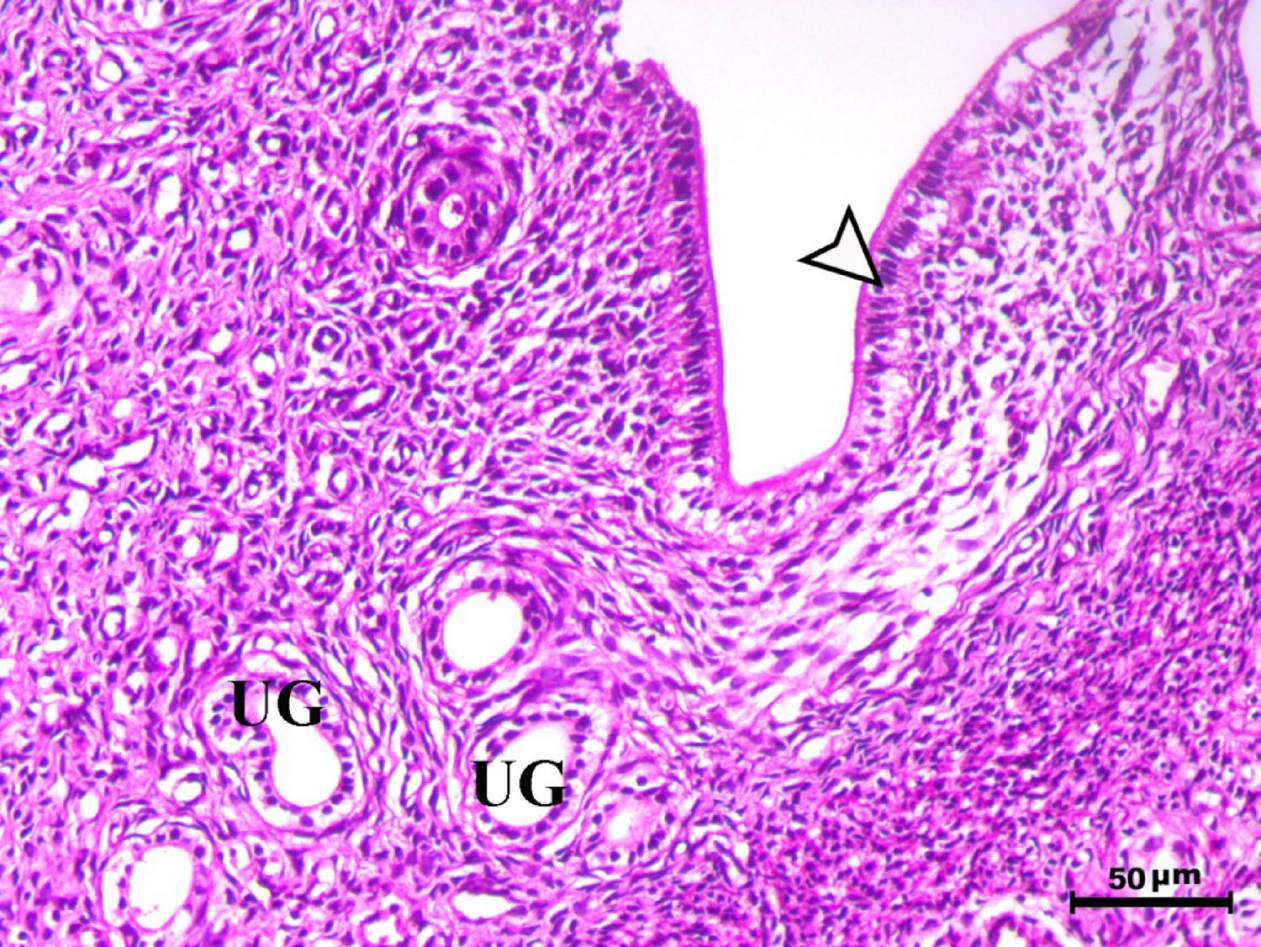
Uterus of control animal G5 showing normal endometrium with normal endometrial lining (arrowhead) and normal endometrial glands (UG), H&E stain, bar = 50 µm

Histopathological analysis of the uterus revealed significant changes between the control, infected, and treated groups. The control groups (Figs. 1, 4, 5, and 6, G1, G3, G4, and G5) exhibited normal uterine structure with intact endometrial lining epithelium (arrowhead) and well-formed endometrial glands (UG), indicating healthy tissues. In contrast, the infected group (Figs. 2 and 3, G2) displayed notable pathological changes, including catarrhal endometritis with hyperplasia of the endometrial epithelium and the presence of Toxoplasma cysts (Fig. 2, arrowhead). Additionally, granuloma-like lesions with prominent mononuclear cell infiltration were observed within the endometrial mucosa (Fig. 3), reflecting severe inflammatory damage caused by *T. gondii* infection.

**Fig. 7:**
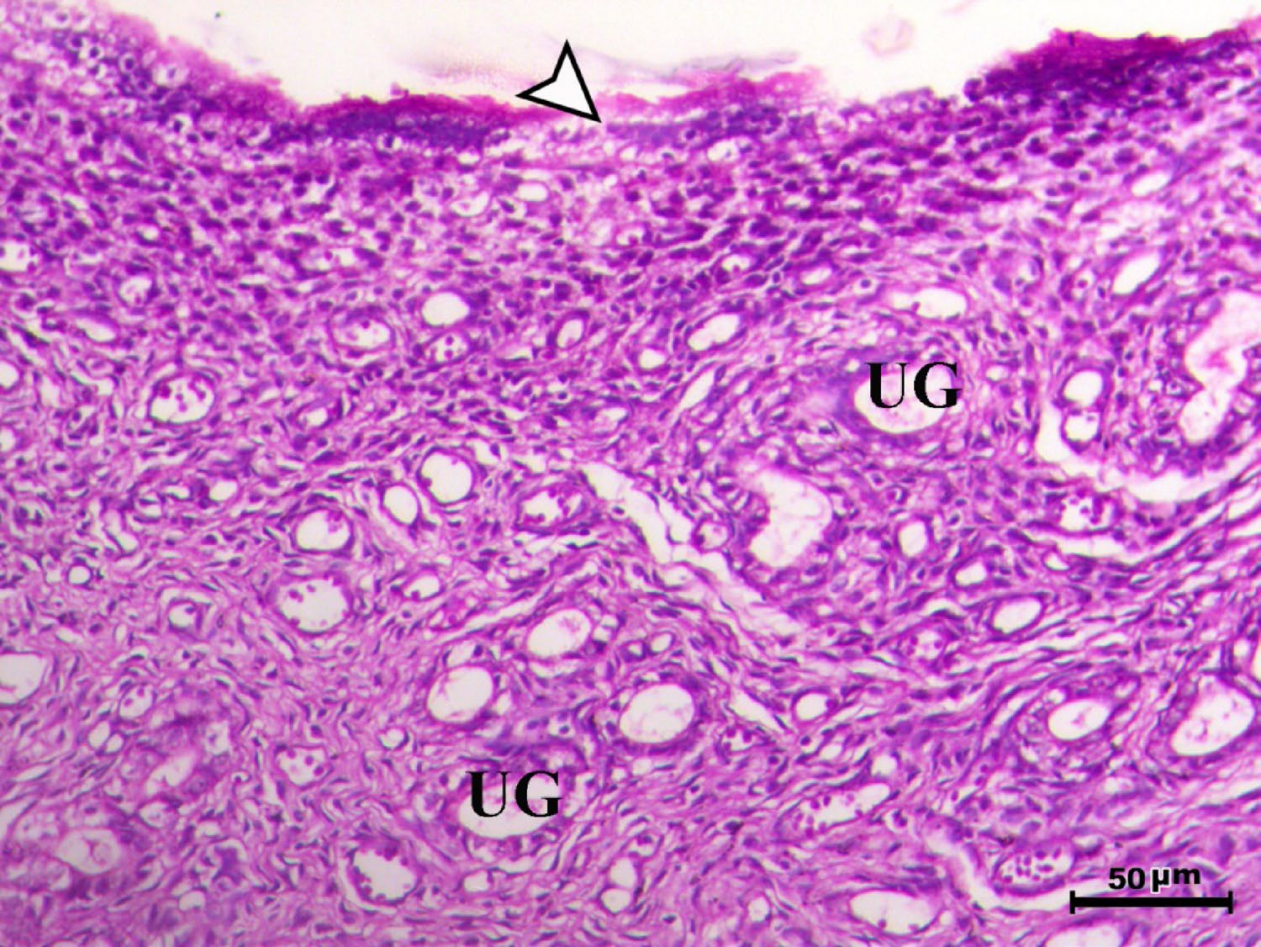
Uterus of diseased animal treated with Spiramycin G6 showing mild catarrhal changes associated with slight desquamation of the lining mucosa (arrowhead) and normal endometrial glands (UG), H&E stain, bar = 50 µm

**Fig. 8:**
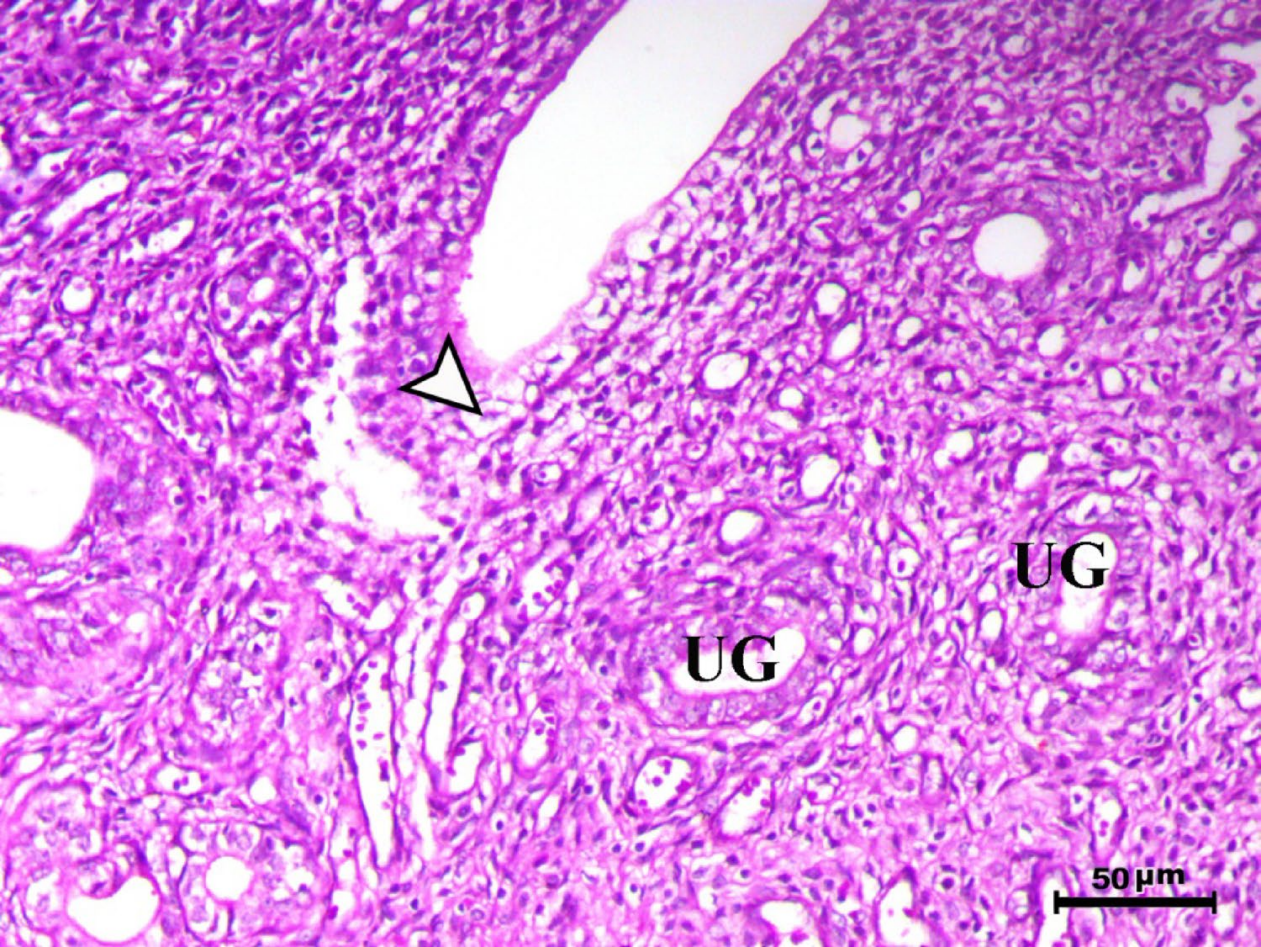
Uterus of diseased animal treated with S-methylcysteine G7 showing mild catarrhal changes associated with mild vacuolation of the lining epithelium (arrowhead) and normal endometrial glands (UG), H&E stain, bar = 50 µm

**Fig. 9:**
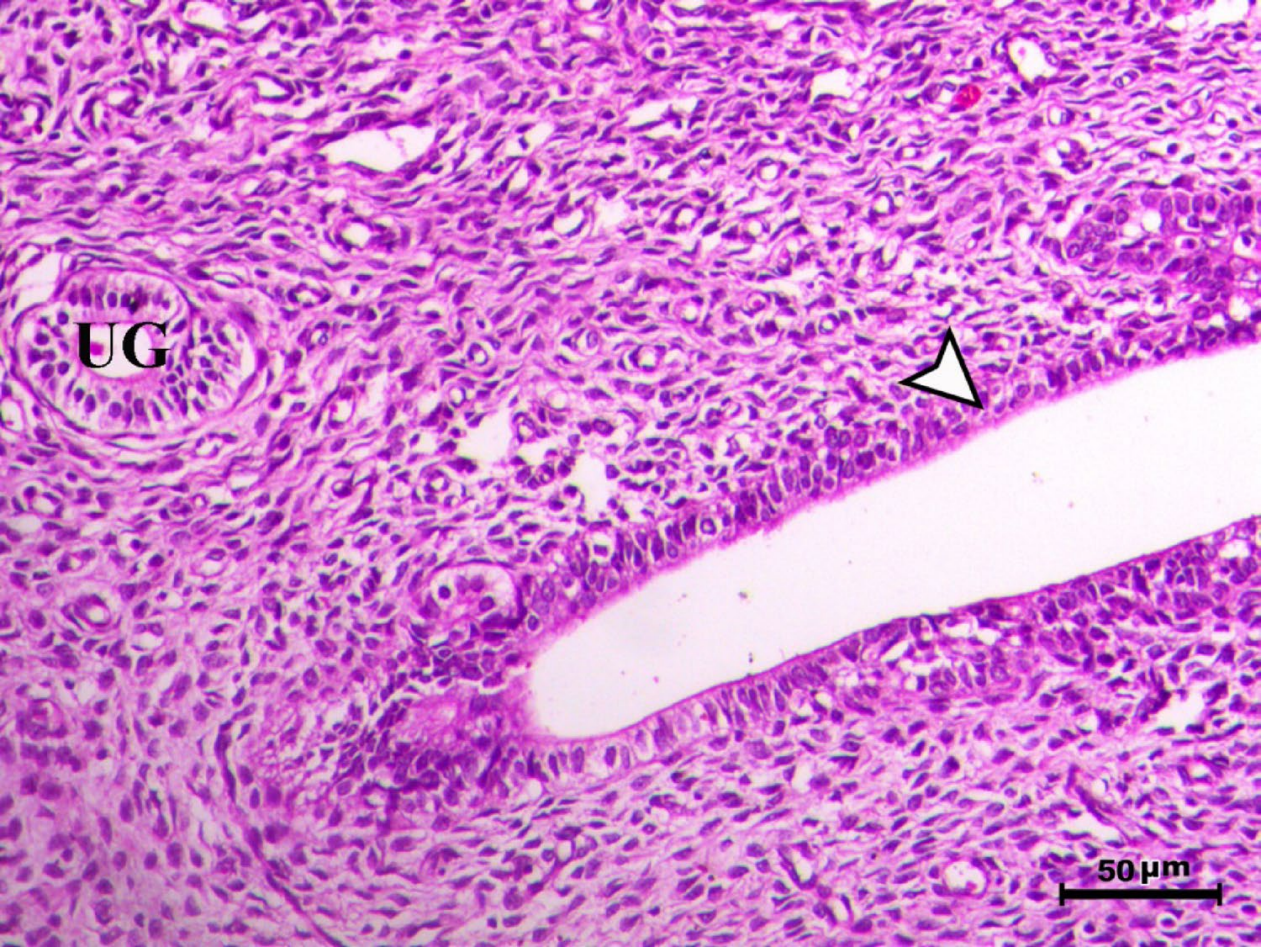
Uterus of diseased animal G8 treated with combined treatment (Spiramycin + S-methyl cystin) showing normal endometrial lining (arrowhead) and normal endometrial glands (UG), H&E stain, bar = 50 µm

Treated groups demonstrated varying degrees of recovery. In groups treated with Spiramycin Fig. 7(G6), mild catarrhal changes with slight desquamation of the lining mucosa were noted, while endometrial glands remained normal. In groups treated with S-methylcysteine Fig. 8(G7), the uterine tissues showed mild catarrhal changes with vacuolation of the lining epithelium (arrowhead) but otherwise retained normal glandular structure. Notably, combined treatment group Fig. 9(G8) exhibited a fully recovered uterus with normal endometrial lining and intact endometrial glands, indicating near-complete healing following treatment.

### Histopathological Examination of the Ovary in Control, Infected, and Treated Groups

**Fig. 10:**
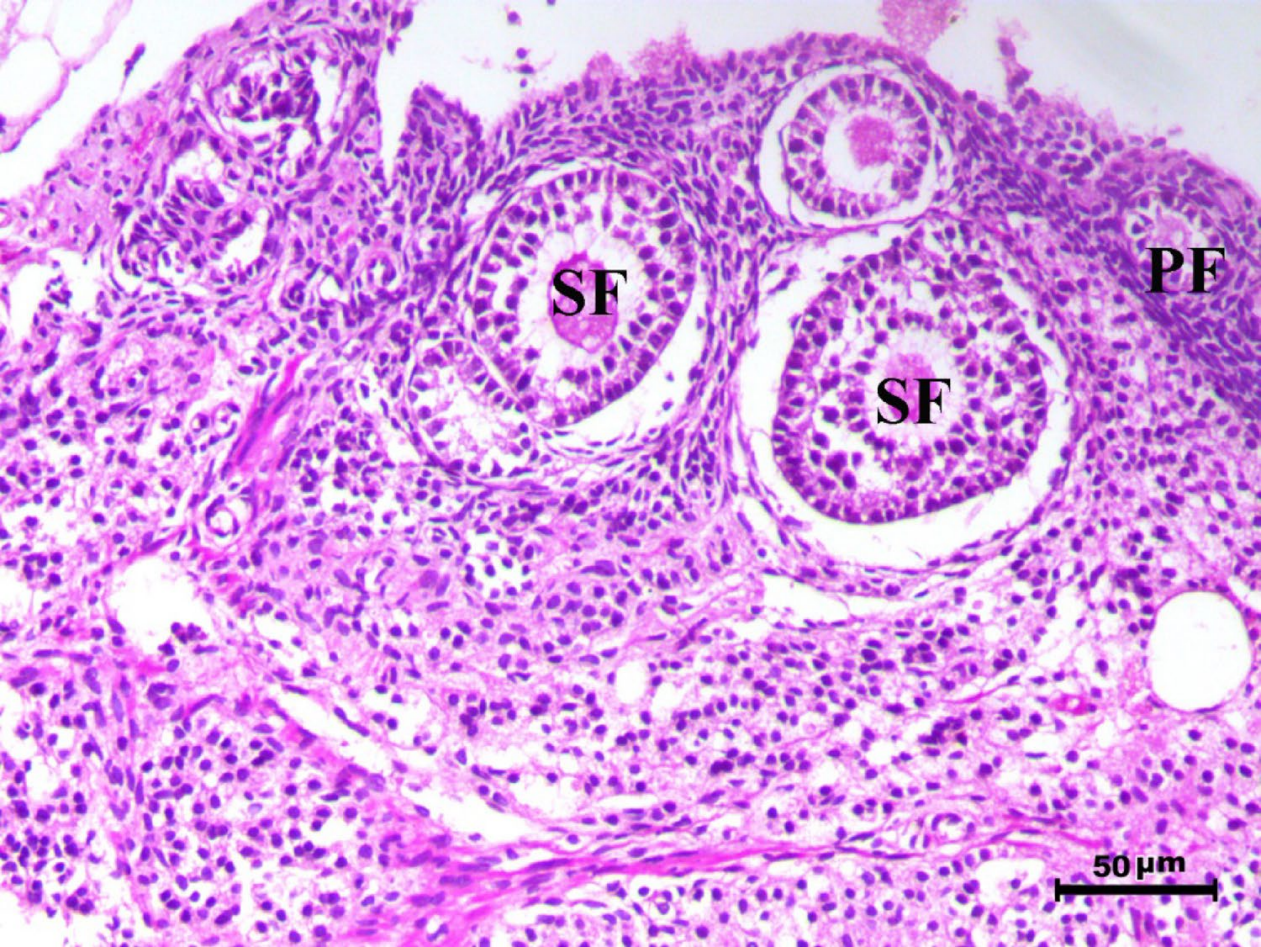
Ovary of G1 animal showing normal cyclic ovary with multiple growing primary and secondary follicles (PF indicates primary follicle and SF indicates secondary follicles), H&E stain, bar = 50 µm

**Fig. 11:**
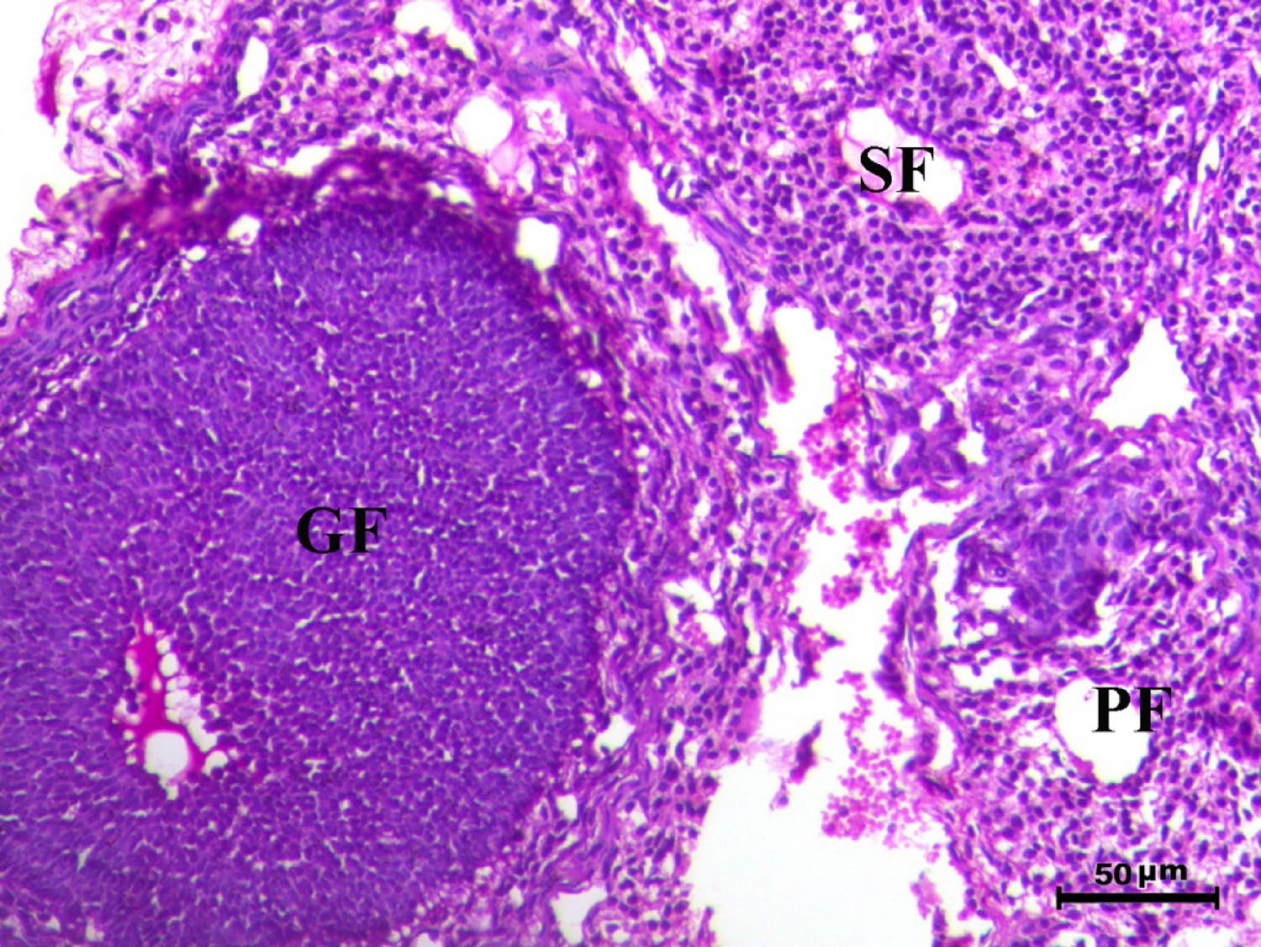
Ovary of diseased animal (G2) showing severe degree of obliterative follicular atresia within the primary, secondary and graafian follicles associated with complete degeneration of the oocytes (PF indicates primary follicle, SF indicates secondary follicles and GF indicates graafian follicles), H&E stain, bar = 50 µm

**Fig. 12:**
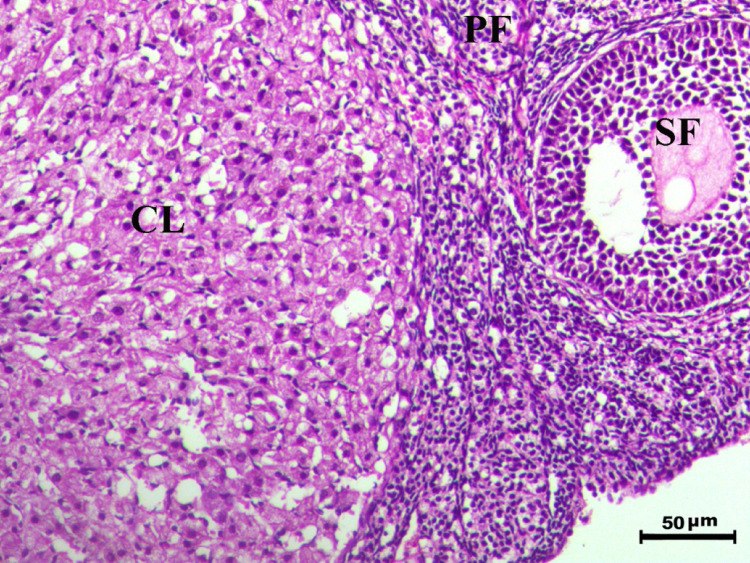
Ovary of G2 animal showing normal cyclic ovary with normal primary (PF), secondary follicle (SF) and normal corpus luteum (CL), H&E stain, bar = 50 µm

**Fig. 13:**
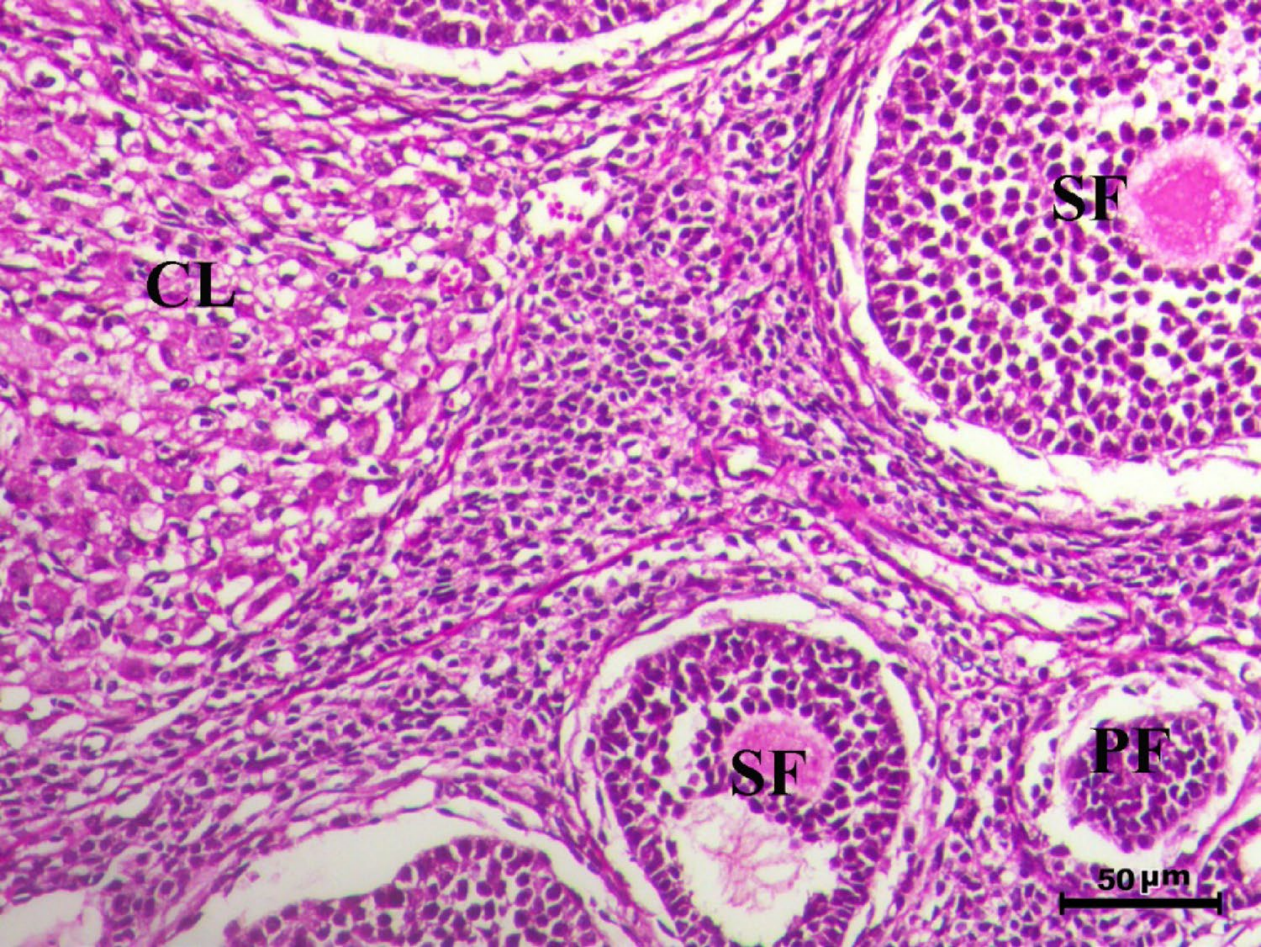
Ovary of G4 animal showing normal cyclic ovary with multiple growing primary (PF), secondary follicle (SF) and normal corpus luteum (CL), H&E stain, bar = 50 µm

**Fig. 14:**
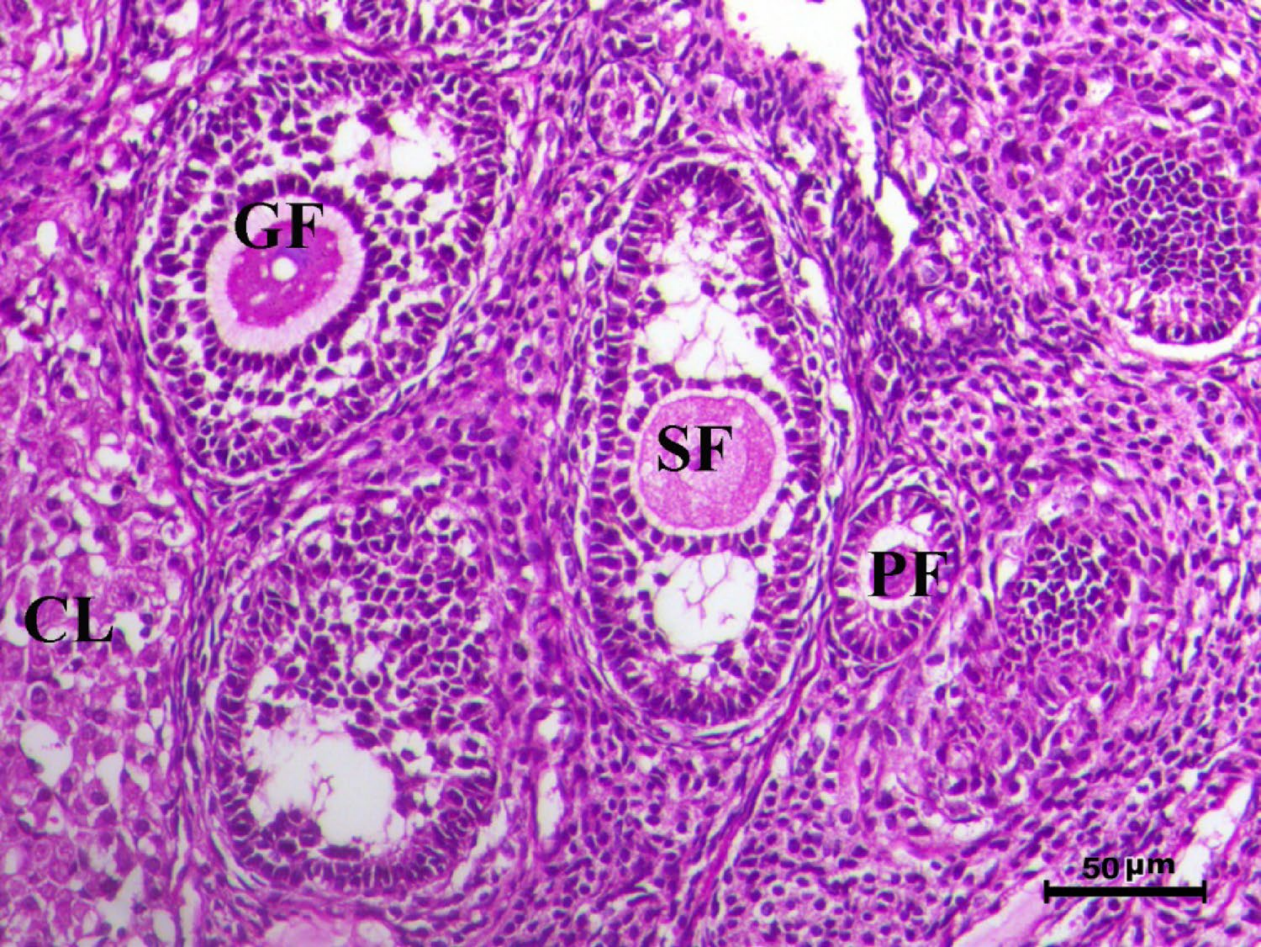
Ovary of G5 animal showing normal cyclic ovary with multiple growing primary (PF), secondary (SF) and graafian (GF) follicles and normal corpus luteum (CL), H&E stain, bar = 50 µm

The histopathological analysis of the ovaries highlights significant variations among control, infected, and treated groups. The control groups (Figs. 10, 12, 13, and 14, G1, G3, G4, and G5) displayed normal ovarian architecture with multiple growing primary (PF) and secondary follicles (SF), as well as normal Graafian follicles (GF) and corpus luteum (CL), indicating active folliculogenesis and normal cyclic function. In contrast, the infected group (Fig. 11, G2) showed severe pathological changes, including obliterative follicular atresia affecting primary, secondary, and Graafian follicles, with complete degeneration of oocytes, reflecting the detrimental impact of *Toxoplasma gondii* infection on ovarian function.

**Fig. 15:**
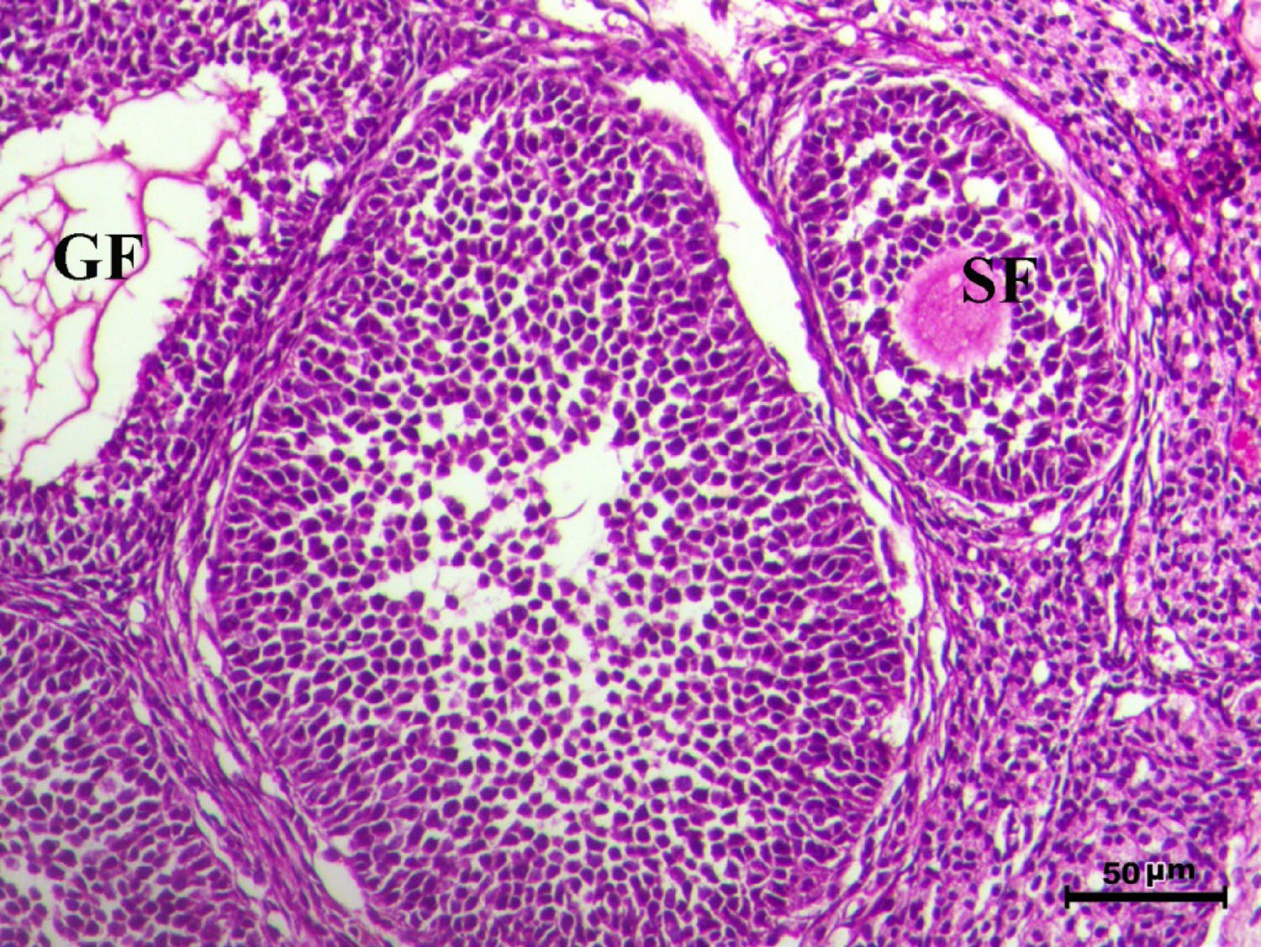
Ovary of G6 animal showing increase the number of the growing follicles and decrease follicular atresia (PF indicates primary follicle, SF indicates secondary follicles, and GF indicates graafian follicles) H&E stain, bar = 50 µm

**Fig. 16:**
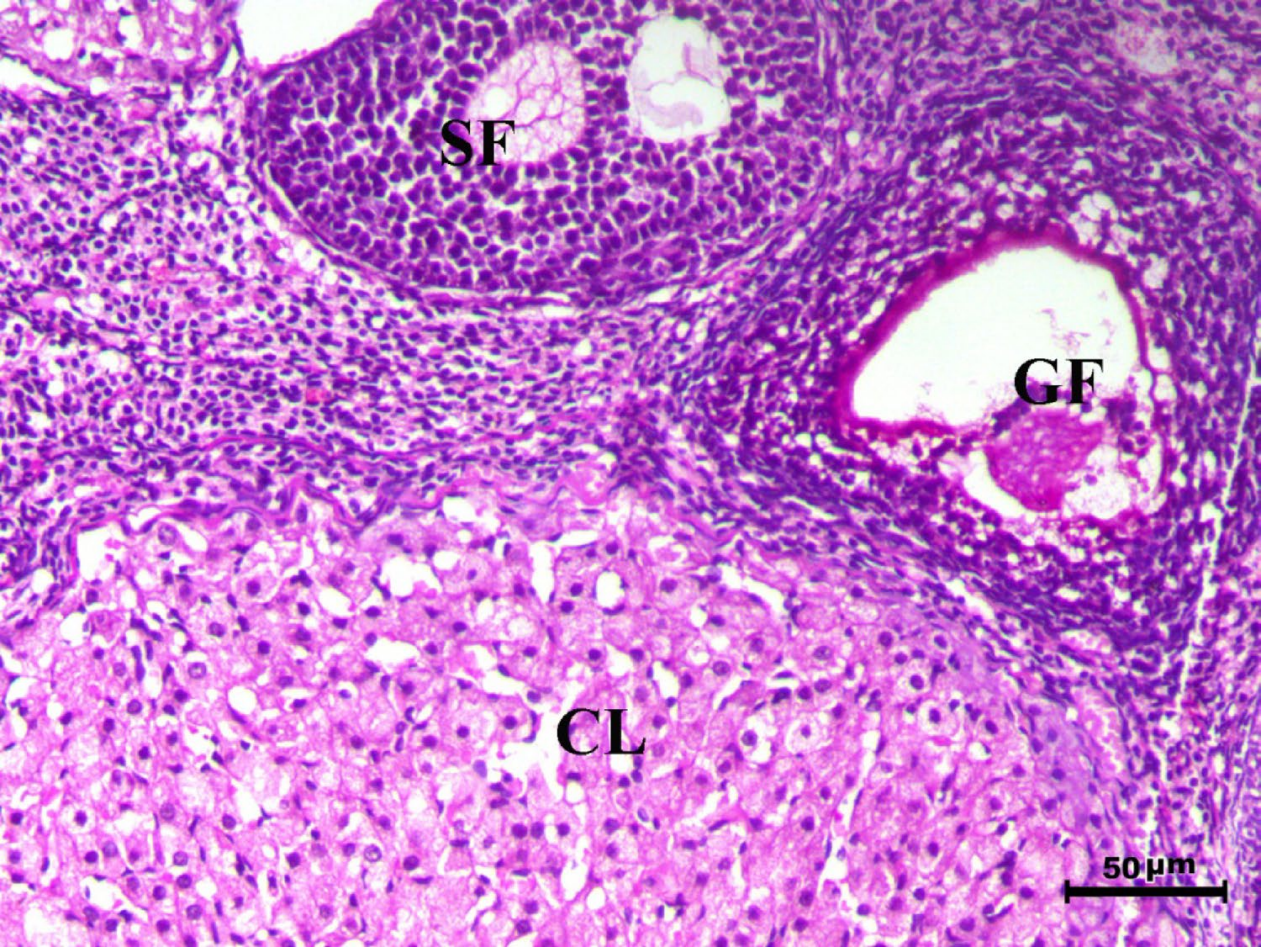
Ovary of G7 animal showing decrease the atretic follicles with increase the number of the growing follicles including secondary follicle (SF), normal corpus luteum (CL) and with only atresia of the graafian follicles (GF) and, H&E stain, bar = 50 µm

**Fig. 17:**
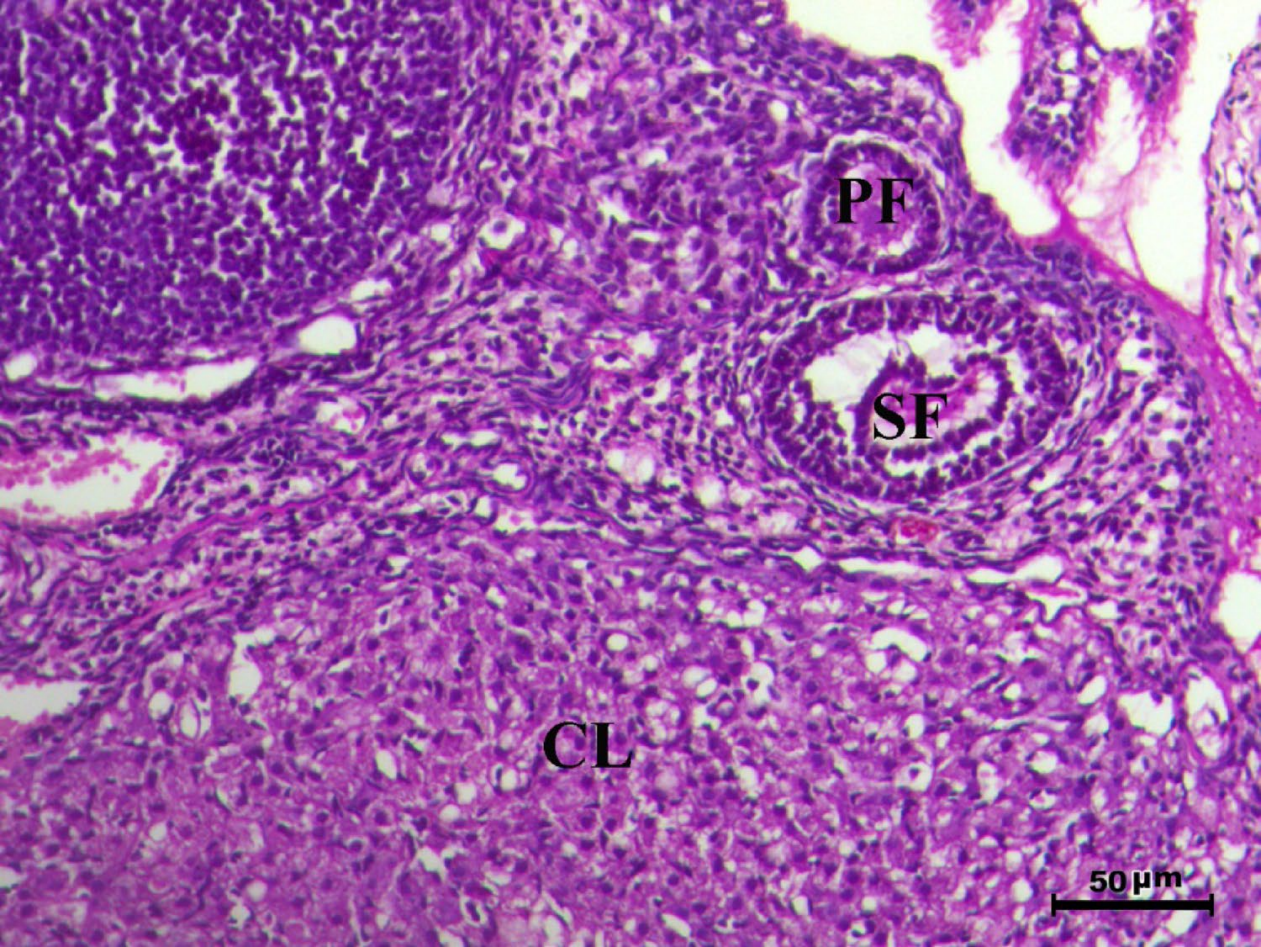
Ovary of G8 animal showing normal folliculogenesis associated with increase the number of the growing follicles containing of primary (PF) and secondary follicles (SF) and corpus luteum (CL), H&E stain, bar = 50 µm

The treated groups exhibited various degrees of recovery. Figure 15(G6) treated with Spiramycin demonstrated an increase in the number of growing follicles and a decrease in follicular atresia, indicating partial restoration of ovarian function. Figure 16(G7) treated with S-methylcysteine showed reduced atretic follicles, increased growing follicles (particularly secondary follicles), and normal corpus luteum, with atresia restricted to Graafian follicles, suggesting moderate improvement. Notably, combined treatment group Fig. 17(G8) revealed near-complete restoration of ovarian function, characterized by normal folliculogenesis, increased numbers of growing follicles (PF and SF), and a well-developed corpus luteum, highlighting the effectiveness of the combined treatment.

## Discussion

This study provides crucial insights into the detrimental effects of *Toxoplasma gondii* infection on the hormonal balance and reproductive health of female rats, further elucidating the complex interplay between parasitic infection and endocrine disruption. Importantly, we demonstrate the therapeutic potential of S-methyl cysteine (SMC) in mitigating these disruptions, offering a potential avenue for managing the reproductive consequences of toxoplasmosis. Our findings highlight the significant ameliorative effects of SMC, particularly when administered in combination with Spiramycin, on both hormonal profiles and histopathological integrity of reproductive organs.

Our study revealed that *T. gondii* infection in female albino rats leads to remarkable hormonal disruption, manifested by high serum estrogen and progesterone levels, accompanied by reduced follicle-stimulating hormone (FSH) and luteinizing hormone (LH). Simultaneously, infected rats showed severe histopathological damage in both the uterus and ovaries, including endometritis, granuloma-like lesions, and extensive follicular atresia. Treatment with S-methylcysteine (SMC) alone alleviated these adverse effects to some extent. However, the most beneficial therapeutic effects were found with the combination of SMC and Spiramycin, which led to restoration of hormonal balance to a near-complete level and significant recovery of uterine and ovarian histopathological structures. These findings highlighted the potential of SMC, especially in combination therapy with conventional anti Toxoplasma drugs as an innovative strategy to oppose *T. gondii*-induced reproductive disruption.

### Hormonal Analysis

#### Hormonal Dysregulation Induced by *T. Gondii*

The findings of the current study support previous studies demonstrating that *T. gondii* infection negatively affects the hormonal balance significantly, which is crucial for reproductive health [30–32]. Recent studies seek to clarify the parasite's complex influence on reproductive functions, such as strain-dependent effects on ovarian health in rat models [25] and even broader implications for male fertility such as adverse effects on human spermatozoa [33].

Specifically, the present study displayed a significant increase in serum estrogen levels in infected rats (137 ± 1.49 pg/ml) compared to the control group (104 ± 2.43 pg/ml) (Table 1). This finding is consistent with previous research which stated that *T. gondii* can affect estrogen synthesis or metabolism either directly or indirectly [1, 2]. Raised estrogen levels are well-recognized to be related to many reproductive dysfunctions and complications, such as irregular menstrual cycles, impaired fertility, and an elevated risk of certain gynecological pathologies [34].

Similarly, progesterone levels were significantly raised in infected rats (12.2 ± 0.338 ng/ml) compared to controls (7.70 ± 0.262 ng/ml) (Table 1). This observation aligns with previous studies showing that *T. gondii* can change progesterone synthesis and metabolism [7]. Abnormal progesterone levels can cause ovulation disruption, affect implantation, and pregnancy maintenance [35]. The simultaneous elevation of both hormones (estrogen and progesterone) suggests a wide disruption of the hypothalamic-pituitary–gonadal (HPG) axis as a result of *T. gondii* infection.

Additionally, *T. gondii* infection resulted in a significant decrease in FSH levels (2.59 ± 0.188 mIU/ml) compared to controls (4.51 ± 0.199 mIU/ml) (Table 1). FSH is well-documented to play an important role in follicular development and oocyte maturation. Decreased FSH levels directly disrupt ovarian function, causing impaired folliculogenesis which might lead to infertility [36]. Similarly, LH levels were significantly decreased in infected rats (9 ± 0.261 mIU/ml) compared to controls (12.0 ± 0.212 mIU/ml) (Table 1). LH is essential for triggering ovulation and facilitating corpus luteum formation [37, 38]. Reduced LH levels can severely impair the normal menstrual cycle and greatly disrupt fertility, underscoring the great influence of *T. gondii* on the whole reproductive endocrine system.

#### SMC Improve Hormonal Homeostasis

Our study reveals the positive therapeutic potential of SMC in reducing the hormonal dysregulation induced by *T. gondii* infection. Treatment with SMC significantly diminished the hormonal imbalances detected in infected rats. Particularly, SMC effectively decreased the raised levels of estrogen and progesterone, about 8% and 13% respectively, compared to the infected group. This demonstrates that SMC may be beneficial in regaining hormonal balance by modifying steroid hormone synthesis or degradation [39], or by decreasing the inflammatory burden that leads to endocrine dysregulation [20].

Additionally, SMC treatment led to a distinct improvement in gonadotropin levels, with substantial rises detected in both LH and FSH. Particularly, LH levels elevated by approximately 12.2%, while FSH levels displayed a more significant rise of about 35.5% compared to the infected group. These hormones play essential roles in follicular development, oocyte maturation, and ovulation [40]. Restoration of these hormone levels suggests potential for ameliorating ovarian function, improving reproductive health and fertility in *T. gondii* infected individuals. This indicates a positive effect of SMC on the HPG axis, possibly by decreasing the negative feedback mechanisms or inflammatory signals inhibiting their secretion during infection.

Remarkably, a synergistic effect was detected when SMC was administered with Spiramycin. The combined therapy showed higher efficacy in regaining hormonal homeostasis than either treatment alone. For estrogen, while both SMC and Spiramycin individually decreased estrogen levels, the combined treatment (I SP SM, 115 ± 2.83 Pg/ml) resulted in the greatest reduction, bringing levels significantly closer to the control group (C, 104 ± 2.43 Pg/ml) than either treatment alone. A similar trend was detected with progesterone; the combined treatment (I SP SM, 9.13 ± 0.305 ng/ml) led to a more pronounced reduction in progesterone levels compared to either SMC or Spiramycin alone, again moving levels closer to the control group (C, 7.70 ± 0.262 ng/ml). For both FSH and LH, the combined treatment resulted in levels that were not only significantly higher than the infected group but also closer to the control group values compared to either individual treatment. This suggests better retrieval of gonadotropin function with the combined therapy. This finding suggests that combining SMC with conventional anti Toxoplasma drugs may improve treatment outcomes and warrants further investigation into the precise mechanisms underlying this synergistic effect. It is probable that SMC's ameliorative effects (e.g., antioxidant, anti-inflammatory) complement Spiramycin's anti-parasitic action, causing a more complete restoration of reproductive function.

### Histopathological Analysis

Besides the hormonal changes, the histopathological examination of the uteri and ovaries offered important evidence of the tissue-level effects of *T. gondii* infection and the improving effects of SMC treatment.

#### Uteri

In control animals, uterine histology appeared normal, with intact endometrial lining and well-organized endometrial glands (Figs. 1, 4, 5, 6). The uteri of infected rats exhibited catarrhal endometritis, characterized by hyperplasia of the endometrial epithelium and the presence of *T. gondii* cysts (Fig. 2). Additionally, granuloma-like lesions with mononuclear cell infiltration were observed (Fig. 3). This finding is in line with previous reports of uterine inflammation and pathology in Toxoplasmosis [1, 2]. *Toxoplasma gondii* infection can trigger a robust inflammatory response in the uterus, leading to tissue damage and impaired function [41]. Such endometritis canseverely compromise reproductive outcomes by impairing embryo implantation, disrupting pregnancy progression, and ultimately contributing to infertility [42, 43].

SMC treatment significantly reduced the severity of endometritis and promoted the restoration of normal uterine morphology (Figs. 7 and 8). The uteri of SMC-treated animals displayed only mild catarrhal inflammation, with slight desquamation of the lining mucosa or mild vacuolation of the lining epithelium, signifying a considerable reduction in inflammation and tissue damage. While both individual treatments (SMC and Spiramycin) showed some improvement in uterine histology, the combined treatment group (Fig. 9) exhibited a fully recovered uterus with normal endometrial lining and intact endometrial glands, indicating near-complete healing.

This substantial enhancement in uterine health indicates that SMC might have significant potential in preventing or treating the reproductive complications related to *T. gondii* infection. SMC has been previously shown to promote tissue repair by enhancing the production of growth factors and stimulating angiogenesis in various models of injury [44]. Furthermore, SMC's well-documented anti-inflammatory properties may directly contribute to resolving inflammation and promoting tissue regeneration, thereby restoring uterine health and function [18].

#### Ovaries

Control rats showed normal ovarian architecture with multiple growing primary (PF) and secondary follicles (SF), also normal Graafian follicles (GF) and corpus luteum (CL), suggestive of active folliculogenesis and normal cyclic function (Figs. 10, 11, 12, 13, 14). On the contrary, the infected group showed severe obliterative follicular atresia, especially influencing primary, secondary, and Graafian follicles, along with complete degeneration of oocytes (Fig. 11). These findings unequivocally reveal the negative impact of *T. gondii* on ovarian function and structure, align with literature on how systemic infections disrupt reproductive health [8, 45]. Follicular atresia, a process of follicular degeneration, can cause a substantial decrease in viable oocyte production and eventually lead to infertility [46]. This finding is consistent with previous studies showing that *T. gondii* infection can lead to follicular degeneration and ovarian dysfunction [34, 35].

SMC treatment decreased the extent of follicular atresia significantly and improved the growth of healthy follicles (Figs. 15 and 16). The ovaries of SMC-treated animals displayed an increase in growing follicles and a decrease in atretic follicles, demonstrating a considerable restoration of normal folliculogenesis. This observation suggests that SMC may help to improve ovarian function and fertility in *T. gondii* infected individuals. The combined treatment group (Fig. 17) showed the most substantial improvement of ovarian function, as demonstrated by increased numbers of growing follicles (PF and SF), normal folliculogenesis, and a well-developed corpus luteum. This comprehensive normalization highlights the superior efficacy of the combined therapeutic approach.

The histopathological analysis generally showed that SMC effectively moderates the negative effects of *T. gondii* infection on both the uterus and ovaries, enhancing tissue repair and renewal of normal function and morphology in these important reproductive organs. These histopathological enhancements are in consistent with the detected hormonal changes, indicating that SMC may help to improve uterine and ovarian function, thereby significantly alleviating the reproductive consequences of *T. gondii* infection. The repair of tissue function and integrity also suggests the rationale of SMC as a therapeutic agent in improving toxoplasmosis-induced reproductive disorders.

### Potential Mechanisms of Action

The mechanisms by which SMC employs its positive effects are likely complex, involving an intricate interplay of antioxidant, anti-inflammatory, and immunomodulatory actions. These properties contribute to modifying the harmful effects of *T. gondii* infection on reproductive health.

#### Antioxidant Effects

*Toxoplasma gondii* infection is known to induce substantial oxidative stress within host tissues, that lead to the production of reactive oxygen species (ROS) which might disrupt signaling pathways, destroy cellular components and impair organ function [47]. This oxidative stress is especially damaging to sensitive reproductive tissues, leading to hormonal imbalances and histopathological damage. S-Methylcysteine is a potent antioxidant, and in this study its therapeutic efficacy is suggested to be mediated through its ability to neutralize this oxidative stress [18, 48]. SMC may exert its antioxidant effects through several mechanisms: it can augment the activity of endogenous antioxidant enzymes such as catalase (CAT), superoxide dismutase (SOD), and glutathione peroxidase (GPx), which are important for cellular defense against oxidative stress; it can directly scavenge free radicals, thus preventing their damaging effects; and it can contribute to the preservation of intracellular glutathione levels, a key component of the antioxidant defense system [49]. By reducing oxidative stress, SMC helps preserve hormonal homeostasis, maintain cellular integrity, and protect against the cellular and tissue impairment observed in the reproductive organs during *T. gondii* infection.

#### Anti-Inflammatory and Immunomodulatory Effects

*Toxoplasma gondii* infection triggers a strong inflammatory response in the host, which, while important for parasite control, can also lead to tissue pathology and endocrine disruption if dysregulated [41]. The detected endometritis and granuloma formation in infected rats are strong indicators of this inflammatory process. SMC has substantial anti-inflammatory properties that may play an important role in mitigating these effects [18]. SMC can modify the immune response by modulating the production and activity of several inflammatory mediators. For instance, it may inhibit the release of proinflammatory cytokines such as Tumor Necrosis Factor-alpha (TNF-α), Interleukin- beta (IL- β), and Interleukin-6 (IL-6), which are known to promote hormonal disruption and tissue damage during infection [15, 50]. By reducing excessive inflammation, SMC helps to restore the physiological environment critical for normal hormonal function and enhances tissue repair in the reproductive organs, thereby decreasing the severity of histopathological lesions.

#### Direct Effects on Hormone Regulation

While the antioxidant and immunomodulatory effects of SMC are likely to have an important role in its therapeutic benefits, it is also possible that SMC directly affects hormone regulation by binding with hormone receptors or signaling pathways. Emerging evidence suggests that organosulfur compounds, like SMC, can modulate the expression of genes involved in steroidogenesis and hormone signaling [17, 44]. For instance, SMC might affect the interaction of hormones to their receptors or change the activity of key enzymes participated in hormone synthesis or metabolism. This direct interaction could contribute to the detected normalization of estrogen, progesterone, FSH, and LH levels.

### Synergistic Effects and Clinical Implications

Our study detected a synergistic effect when SMC was combined with Spiramycin. This finding has essential implications for treatment strategies, signifying that combining SMC with conventional anti-Toxoplasma drugs, such as Spiramycin which is still used in clinical settings for certain patient populations, may improve treatment outcomes and enhance reproductive health in *T. gondii* infected individuals.

The detected synergism is likely due to the combined complementary actions of the two compounds. Spiramycin, an antibiotic, primarily acts by inhibiting protein synthesis in the parasite, thereby decreasing parasitic burden [12]. However, it can have side effects and has limited efficacy against the latent bradyzoite stage. On the other hand, SMC, through its antioxidant, anti-inflammatory, and potentially immunomodulatory properties, mitigates the host-mediated pathology induced by the infection, such as oxidative stress and inflammation, which participate in hormonal disruption and tissue damage. By concurrently targeting the parasite (Spiramycin) and modulating the host response pathology (SMC), the combined therapy offers a more effective and comprehensive approach to managing toxoplasmosis and its reproductive consequences.

Clinically, SMC has encouraging potential as a therapeutic agent for treating the reproductive sequalae of toxoplasmosis, especially in women. It could be administered as a combined therapy with other traditional drugs like Spiramycin or pyrimethamine/sulfadiazine. This could enhance treatment outcomes by improving efficacy, reducing side effects by limiting the duration or dosage of conventional drugs, and achieving a more comprehensive recovery of reproductive function. This is particularly important for women experiencing reproductive complications due to *T. gondii* infection like infertility, recurrent pregnancy loss. Moreover, considering the substantial risk of congenital transmission, the beneficial effects of SMC on maternal reproductive health might play an indirect, yet important role in preventing negative pregnancy outcomes related to toxoplasmosis, although this would necessitate particular investigation. The results suggest a shift towards combination therapies that target the parasite itself and also enhance host recovery and ameliorate disease-induced pathology. This holistic approach could present a safer and more effective treatment regimens for toxoplasmosis, a prevalent and often debilitating parasitic disease.

While S-methylcysteine shows therapeutic promise, its potential off-target effects and toxicity, especially with long-term use, require careful consideration. High doses have been linked to cardiotoxicity in animal models, and related compounds have shown toxicity in some species [28]. Although human data on SMC toxicity is limited, general side effects of cysteine derivatives can include gastrointestinal issues, headaches, and more severe reactions like allergic responses or liver problems [20]. The current study, conducted on rats with a specific dosage and duration, did not observe overt toxicity. However, further comprehensive toxicological assessments, including dose–response and long-term safety studies, are essential to establish safe and effective clinical regimens for SMC.

### Future Directions

Future research should focus on three key areas to build upon this study's findings. First, the exact molecular and cellular mechanisms of S-Methylcysteine (SMC) need to be elucidated, including its effects on hormonal pathways, inflammation, and antioxidant systems, potentially using techniques like transcriptomics and proteomics. Second, combination therapy protocols should be optimized by exploring different dosages and ratios of SMC with Spiramycin and other anti-Toxoplasma drugs against various parasite strains and infection stages. Finally, the promising results from this rat model must be validated in other animal models and, ultimately, in well-designed human clinical trials to confirm the safety, efficacy, and optimal dosing of SMC for treating toxoplasmosis-related reproductive issues.

### Limitations of the Study

It is important to acknowledge several limitations of this study. First, the findings are from a rat model and may not be directly transferable to humans due to physiological differences. Second, while potential antioxidant and anti-inflammatory properties were proposed, the precise molecular mechanisms of SMC were not fully identified. Lastly, this research focused exclusively on female reproductive toxicity; the potential effects of *T. gondii* and SMC on male reproductive health were not explored. Addressing these limitations in future research will provide a more complete understanding of SMC's therapeutic potential.

## Conclusion

The present study demonstrates the promising potential of SMC as a therapeutic agent for mitigating the hormonal disruptions, histopathological damage, and reproductive consequences associated with *T. gondii* infection. Future studies should focus on elucidating the mechanisms of action, exploring the efficacy of combination therapies, and conducting clinical trials to assess the safety and effectiveness of SMC in human patients. Continued research will pave the way for its broader clinical application and contribute significantly to global health efforts against this prevalent parasitic disease.

## Data Availability

Data is provided within the manuscript
